# EFFORT-D: results of a randomised controlled trial testing the EFFect of running therapy on depression

**DOI:** 10.1186/s12888-019-2156-x

**Published:** 2019-06-10

**Authors:** Frank Kruisdijk, Marijke Hopman-Rock, Aartjan T. F. Beekman, Ingrid Hendriksen

**Affiliations:** 1 GGz Centraal Innova, Amersfoort, the Netherlands; 2 Body@Work, TNO-VU University Amsterdam Medical Center, Amsterdam, the Netherlands; 3 The Netherlands Organisation for applied scientific research TNO, Leiden, the Netherlands; 4 Amsterdam University Medical Center, location VUmc, Department of Psychiatry, Amsterdam, the Netherlands; 5 Department of Public and Occupational Health, Amsterdam Public Health Research Institute, Amsterdam University Medical Center, Amsterdam, the Netherlands

**Keywords:** Major depressive disorder, Specialized mental health care, Exercise, Submaximal bicycle test, Cardio-metabolic risk

## Abstract

**Abstract:**

Results of a randomised controlled trial testing the EFFect Of Running Therapy on Depression.

**Background:**

This randomised controlled trial explored the anti-depressive and health effects of add-on exercise (running therapy or Nordic walking) in patients with Major Depressive Disorder (MDD).

**Methods:**

Patients were recruited at three specialised mental health care institutions. In the intervention group exercise was planned two times a week during 6 months, the control group received care as usual. Observer-blinded measurements included Hamilton-17 depression scores and several health and fitness parameters. Submaximal bicycle-tests were performed at inclusion, 3, 6 and 12 months. The effects of exercise were assessed by effect size, intention-to-treat and analysis per protocol using General Linear Models (GLM) with time x group interactions.

**Results:**

In total, 183 patients were assessed for eligibility and 135 were excluded (40% of the potential participants declined to participate mainly due to a lack of time and motivation). Together with a drop-out of 55% at 6 months, this reduced the power of the study severely. As a result, statistical analysis was performed only on the first 3 months of the study. Data were ultimately analysed from 46 patients, of which 24 were in the intervention group. Significantly more women were in the intervention group, and depression and fitness were higher in the control group. Participants showed 2–3 points less depression on average after 3 months. However, the GLM showed no effect on depression (*Cohen’s d < 0.2, F = .13, p = .73)* in both the intention-to-treat and per protocol analyses. However, large effect sizes (*Cohen’s d > 0.8*) were found for aerobic capacity (*VO*_*2*_*max∙.kg*^*− 1*^, F = 7.1, p = .02*), maximal external output (*Wmax∙.kg*^*− 1*^, F = 6.1, p = .03*), and Body Mass Index (F = 5, p = .04*), in favour of the intervention group.

**Conclusions:**

In this selective and relative small clinical population with MDD, an anti-depressive effect of the exercise intervention could not be measured and is also unlikely due to the very low effect size. An integrated lifestyle intervention will probably be more effective than a single add-on exercise intervention. However, significantly increased fitness levels may contribute to the alleviation of current cardio-metabolic risk factors or prevention of these in the future.

Trial registration: Netherlands Trial Register (NTR): NTR1894 on July 2nd 2009.

## Background

Major Depressive Disorder (MDD) is a leading cause of disability and loss of quality of life worldwide [1]. Depression has complex, reciprocal associations with several physical illnesses, including diabetes mellitus and cardiovascular disease [2–5]. A recent body of research suggests that shared immuno-metabolic etiological pathways could explain the comorbidity between depression and metabolic syndrome, diabetes, autonomic nervous system dysregulation and cardiovascular disorders. This idea is based on recent findings in genome-wide association studies (GWAS) of depression [6], clinical epidemiological work [7] and experimental work testing the effects of anti-inflammatory agents in patients with treatment-resistant depression [8]. Antidepressant pharmacotherapy and/or cognitive behavioural (CBT) and interpersonal psychotherapy (IPT) [9] are currently the mainstays of treatment for MDD. Language barriers, costs and availability of therapists could be a barriers to CBT and IPT. Regarding medication, only 30% of the patients reach full remission within 12 weeks using these first-line antidepressant treatments [10]. Medication can also have undesirable side-effects, such as increased cardiovascular risk, and is often resisted by patients, leading to poor adherence and suboptimal response.

As an alternative or add-on, most guidelines on the treatment of depression [11–14] recommend physical activity (PA), such as walking, cycling and exercise (a planned, structured, repetitive and purposive physical activity [15]). The effect of PA on depression has been tested extensively. However, in most randomised controlled trials (RCTs) studying the effect of exercise on depression [16–18], the included patients had mild to moderate depression. This is partly because most of these studies relied on self-selected volunteers and/or patients who were included based on a self-rating depression scale (Becks Depression Inventory [19]). Only some of the participants were diagnosed by a trained clinician with an unipolar depression or MDD according to the Diagnostic and Statistical Manual of Mental Disorders (DSM) [20] or the International Classification of Diseases (ICD) [21]. Three such RCTs studied adult inpatients with MDD (total *n* = 113), including one study that was published in 1985 [22].

Due to ethical considerations when treating patients with MDD, in current clinical practice an add-on approach is preferable to stand-alone exercise treatment. Depression in these patients is often severe and protracted, and they are already using antidepressants. Therefore, when testing the effect of PA in specialised mental health care settings, an add-on design – i.e. adding PA to ongoing treatments – reflects real-world clinical practice better than testing exercise as a monotherapy, which may be appropriate in patients with milder depressive illness. To our knowledge, few recent RCTs [23] have tested the effect of adding PA to ongoing care in clinically diagnosed MDD patients in regular specialised mental health care. To this end, we designed the EFFORT-D study: EFFect Of Running Therapy on Depression. The intervention consisted of running therapy (RT) or Nordic walking (NW) during 6 months and a post-intervention follow-up at 12 months. The study design of EFFORT-D has been published previously [24]. The main objective of the study was to assess the effectiveness of RT or NW, in addition to usual care, on MDD in adult inpatients and outpatients. We hypothesised that adding exercise therapy to usual care would result in a larger reduction in depressive symptoms (as measured with the Hamilton Rating Scale of Depression (HAM-D17, see method section), after a 6 month treatment programme as well as after another 6 months follow-up, compared to usual care without exercise therapy. Secondary objectives were to assess the effectiveness of RT or NW on the following outcome measures: 1) Fitness parameters: maximal oxygen uptake (VO_2_max∙kg^− 1^), maximal external power output (Wmax∙kg^− 1^), heartbeat at rest, grip strength; 2) Metabolic risk factors: body composition (Body Mass Index, fat-free mass, muscle mass, visceral fat, waist circumference), blood pressure, fasting blood glucose, blood fats; 3) Co-morbid symptoms of anxiety and pain; 4) Quality of life; 5) Cost-effectiveness.

## Method

### Design

We conducted an observer-blinded RCT with adult patients with MDD treated in either an inpatient department or in an outpatient clinic or day hospital.

### Setting

The study was carried out between December 2012 and January 2015 in three specialised mental health care institutions in the Netherlands. The setting included patients from two inpatient emergency wards and three specialised outpatient clinics for affective disorders, ensuring the inclusion of patients with more severe, protracted disorders. As a result of unforeseen conditions, which were described previously in a process evaluation study [25], 6 months after the start of the study, one of the specialised mental health care institutions was no longer available as an inclusion location. The two remaining institutions consisted of inpatient units and day hospitals with multidisciplinary teams with psychiatrists, a specialised mental health care general practitioner (GP), a neurologist on demand, psychotherapists, sociotherapists, occupational and physical therapists, a social worker and nurses. Patients were referred by outpatient mental health care services (emergency or otherwise). The patients assigned to two locked emergency wards continued their stay after stabilisation and reduction of suicide risk in an open crisis ward, and often continued their treatment in a day hospital followed by the outpatient programme (psychotherapy, medication and outreaching social nurse).

Patients of the two outpatient clinics were referred by GPs, and were assessed, diagnosed and treated by a psychiatrist, psychotherapist and/or a social psychiatric nurse.

### Participants

Eligible participants were patients between 18 and 65 years old with MDD, bipolar depression or seasonal depression not responding to bright light therapy. Patients with severe and acute symptoms of MDD who were referred to an emergency ward were eligible for inclusion and, when stabilised and capable of giving informed consent, were visited by the research assistant. All participants were diagnosed by a clinician before referral to the study and fulfilled the criteria for MDD according to the Diagnostic and Statistical Manual of Mental Disorders, DSM- IV-TR [20]. In case of a HAM-D17 score [26] ≥ 14, a cut-off score for mild depression, the candidate was invited to participate after the protocol and aims of the study were explained. Exclusion criteria were a depressive disorder as part of a psychotic disorder, schizophrenia, schizoaffective disorder, or obsessive-compulsive disorder or anxiety disorder as primary diagnosis. Also excluded were patients with significant cardiovascular disease or other medical conditions as contra-indication for exercise therapy, walking and/or running such as joint and hip pathology, alcohol/drugs dependence as a primary diagnosis, pregnancy, high suicide risk with treatment on a closed ward, or already being physically active on a regular basis (2 or more times a week at a high-intensity level).

### Trial staff

The assessment of the HAM-D17 at baseline was performed by the research-assistant prior to randomisation. At the other measurement points, the HAM-D17 was measured by one of the three blinded outcome assessors (psychologists). They were all trained before the start of the study by an expert in HAM-D17 testing to reach consensus. As a control measure during the study, HAM-D17 interrater sessions were performed monthly on a patient with MDD (non-participant in the study), and no more than 2 points difference on the HAM-D17 scale between outcome assessors was accepted. If this criterion was not met, additional training was scheduled.

The inclusion procedure was performed by the research assistant, who was trained for this beforehand. The procedure consisted of an eligibility check followed by an appointment for randomisation, online questionnaires, biometrics, blood pressure measurement and a submaximal Åstrand bicycle test. During the first assessment, a lab appointment for blood sample collection was scheduled.

The intervention trainers were a certificated physiotherapist and running therapists who were very experienced in RT and NW as well as in working with patients with severe mental disorders.

### Recruitment

Within the three hospital regions, attention was drawn to the study with flyers for patients and professionals, announcements on the website, and bilateral visits of the principal researcher and research assistant to the heads and therapists of affective disorder programmes and intake-teams of the outpatient departments. The included participants received one gift-voucher worth 25 euros as an incentive for future participation in training and measurement activities.

### Randomisation and masking

**Fig. 1:**
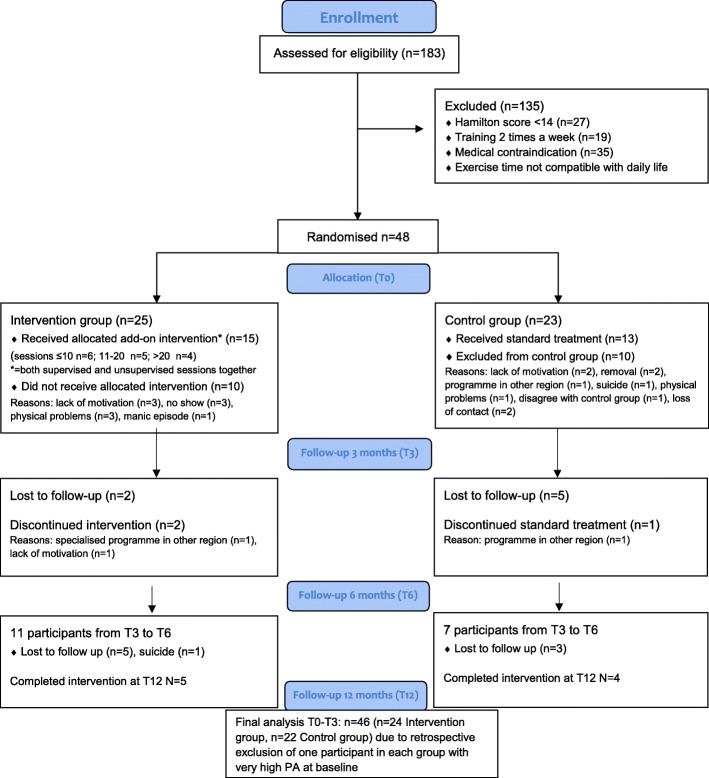
Flow diagram of the EFFORT-D study

Randomisation took place at each location separately. As a result, each location had an equal distribution of participants in the intervention and control group. The SPSS random generator, SPSS version 14.0 [27] was used to allocate patients. Participants were randomly assigned to exercise twice a week for 6 months (the participant could choose NW if RT was not possible, for instance if they had some joint problems), in addition to their treatment as usual or standard treatment. Both groups were followed for 1 year with measurements at inclusion (T0), 3 months (T3), 6 months (T6) and after 12 months (T12). Figure 1 shows the flow diagram of the study.

At the end of the inclusion procedure, if informed consent was given, 10 closed opaque envelopes with allocation numbers were presented to the participants by the research assistant. The participant could choose an envelope, after which the research assistant told the participant in which arm of the study he or she was included, and the participant was enrolled into the study by the research-assistant. Due to the type of intervention, the participants, research assistant and trainers could not be blinded. Evaluators of the main outcome measure (HAM-D17) were blinded for group allocation. All biometric measurements and the Åstrand bicycle test were performed by the research assistant. The questionnaires (described in the measurements subsection) were self-assessed online by digital code and filled in by the participants.

### Intervention

In total, around 40 exercise sessions were scheduled during 6 months. We expected (based on clinical experience) a long remission-to-recovery period in our patient population, from at least 2–3 months. We decided to take a rather long exercise duration of 6 months, because we were also interested in the maintenance of a possible exercise effect which was not described yet in the available literature. These add-on exercise sessions took place twice a week: once a week a supervised group session was offered and once a week the patient exercised individually, with clear instructions on beforehand and an evaluation with self-report at the beginning of the next supervised session. Each supervised session, in which the trainers worked according to a standardised protocol, lasted 1 hour, including 30 min of running therapy (RT) or Nordic walking (NW). The remaining time was spent on warming-up and cooling-down. Each subject followed an individualised intervention protocol with gradually increasing training intensity. The goal was to achieve a 30 min period of continuous running or Nordic walking in the last sessions (30 min of continuous aerobic exercise twice a week at at least 60% of their estimated maximum heart rate). Given the importance of easy access to the training sites, care was taken that both the timing (flexibility of the trainers) and the location (near to the patients and easy to access) were convenient for participants. These intensive exercise sessions were not offered to the control group. They were only allowed to exercise at low intensity (walking and recreational sports) and participate in psycho-motor therapy in their treatment programme. The intervention group also had psycho-motor therapy in the treatment programme and were not encouraged to do intensive exercise between the training sessions. Furthermore, participants in both arms of the study received treatment as usual consisting of pharmacotherapy, cognitive therapy or interpersonal therapy, in addition to psycho-education and lifestyle advice emphasising an active lifestyle with enough walking per day.

### Measurements

All outcome parameters measured during baseline were repeated after three, six and 12 months, except for the blood samples, which were taken only at T0 and T6. [24].

#### Major depressive disorder

The primary outcome was change in severity of depressive symptoms measured with the HAM-D17. This instrument has been shown to have a good interrater reliability of .92 (Pearson) and internal consistency of .82 (Cronbach’s alpha) if used by trained and experienced outcome assessors [28]. It is the most frequently used instrument to measure changes in depressive symptoms in clinical trials, especially in patients with more severe depression [29].

#### Fitness

A submaximal Åstrand bicycle test was performed on a stationary bicycle ergometer (Examiner, Lode BV, the Netherlands). The heart rate during this test was registered with a heart rate monitor (Polar RS 800, Electro Oy, Finland), and the mean heart rate of the last 2 minutes of the test combined with the submaximal workload was used to estimate the maximal external power output (Wmax) and the maximal oxygen uptake (VO_2_max). The estimated Wmax in Watt and VO_2_max in ml∙min^− 1^, both related to body weight (Wmax∙kg^− 1^; VO_2_max∙kg^− 1^), were used as outcome measures.

• The heartbeat at rest was measured by the heart beat monitor.

• Grip-strength was tested according to protocol twice in both hands (highest value was registered) using a hydraulic hand dynamometer (Jamar J00105, Sammons Preston Rolyan, Bolingbrook, USA).

#### Metabolic parameters

• A bio-impedance scale was used to measure weight and body composition (Omron HBF-510, Omron Healthcare Europe BV, the Netherlands). Height was measured according to protocol (Seca 214, Hamburg, Germany) and BMI was calculated by standard formula. Waist circumference was measured twice with a tape measure (Seca 201, Hamburg, Germany) at the midpoint between the lower border of the ribs and the upper border of the pelvis.

• Systolic and diastolic blood pressure were measured twice at rest using an electronic blood pressure meter (Omron M6 comfort, Omron Healthcare Europe BV, the Netherlands) with an adequate cuff size. The mean value of both measurements was registered.

• Blood samples were taken at T0 and T6 to determine fasting blood glucose and blood fat. All blood samples were kept frozen and analysed together in one laboratory session at the end of the study.

#### Psychological and social measurements

• Co-morbid symptoms of anxiety were measured by the Beck Anxiety Inventory (BAI), a 21-item multiple-choice self-report inventory that measures the severity of generalised anxiety and panic symptoms in adults and adolescents on four levels. The BAI has been shown to be highly internally consistent (Cronbach’s alpha = .94) and was scored as sufficient on tests of convergent and discriminant validity [30].

• Co-morbid pain was measured with the Graded Chronic Pain Scale (GCPS), a 7-item scale measuring aspects of pain, physical ability and social interference, resulting in a 5-class hierarchical scale ranging from 0 (no pain problem) to IV (high disability/severely limiting). (Internal consistency in Cronbach’s alpha: .71–.74, Pearson’s correlation coefficient .45–.58).

• Quality of life (QoL) data were collected using the EuroQuol 5 dimension questionnaire (EQ-5D), a standardised instrument that measures five dimensions of health (one item each): mobility, self-care, usual activities, pain/discomfort and anxiety/depression, rated from 1 (no problems) to 3 (many problems). The added value of the EQ-5D is the calculation of an index score ranging from 0 (worst QoL) to 1 (perfect QoL) using the Dutch value set based on time-trade-off weightings of a representative Dutch sample [31].

• Cost-effectiveness: Healthcare use and work productivity were evaluated by the Trimbos/iMTA Questionnaire for Costs associated with Psychiatric Illness (TIC-P), a 29-item list which focuses on establishing costs related to loss of productivity at work and to health care utilisation. A feasibility and validation study showed that the TIC-P is a valid instrument for measuring medical consumption and productivity losses [32].

#### Other variables

##### Physical activity

• SQUASH: This questionnaire measures physical activity (days, time and effort of PA) in four domains of daily life during a normal week in the past months and enables computation of the intensity of energy expenditure. Intensity is defined as the ratio of work metabolic rate to a standard resting metabolic rate (MET) [33]. The SQUASH has been shown to have a Spearman’s correlation coefficient of .58 for total reproducibility and .45 for total relative validity compared with an accelerometer, and is suitable for population studies [34]. This questionnaire was only used retrospectively to determine whether patients were too physically active at T0 and eventually remove them before statistical analysis.

##### Medication

• Type and duration of medication was derived from patient records retrospectively.

### Sample size

It was expected that patients in the usual care group (control group) would respond with a mean reduction in HAM-D_17_ of six points, because they were all following the standard MDD treatment protocol (this was based on clinical experience). Adding exercise to usual care (intervention group) was expected to result in a decline of at least eight points (2 points more than the control group) on the HAM-D_17_ score. To detect this difference, with an α (two-tailed) of 5% and a power (1-β) of 80%, using two equal groups and a standard deviation of 5 points, 100 patients were needed in each group. Taking 30% drop-out into account, 140 patients had to be included in each group [24].

### Data-analysis

The statistical analysis was performed using SPSS version 24 [35]. The normality and homogeneity of the data were tested and confirmed by Kolmogorov-Smirnov tests. Independent Chi^2^-tests and Fisher’s exact (Fe) tests (categorical variables) and analysis of variance (ANOVA) (continuous variables) were used for comparison of the intervention and control group characteristics at baseline. Student’s T-tests with 95% confidence interval were used for independent samples. Intention to treat (ITT) analysis was performed using General Linear Models (GLM) repeated measures with time x group interactions to test the differences in intervention and control group at different times [36]. For the per protocol (PP) analysis, the intervention participants were selected and divided into three groups based on the number of sessions they participated (0–10, 10–20 sessions and > 20 sessions). Participants with more than 10 sessions were included.

### Ethical approval

The EFFORT-D study was reported according to the Consolidated Standards of Reporting Trials (CONSORT) statement [37]. It was designed and implemented in accordance with the principles of the Helsinki Declaration (Edinburgh, Scotland Amendment, October 2000). The study protocol was approved by the Medical Ethical Committee for Mental Health (Metigg Kamer Noord), CCMO (Central Committee on Research Involving Human Subjects) protocol number: NL.26169.097.08.

The EFFORT-D study was registered in the Netherlands Trial Register (NTR): NTR1894 on July 2nd 2009. All participants gave their informed consent for participation in the study.

## Results

### Participant flow during the study

In total 183 patients were assessed as eligible to participate within the 2-year time-frame of the EFFORT-D study.

A relatively high number (*n* = 135) of these eligible participants were excluded for various reasons, see Fig. 1. The largest group of excluded patients (40%) were those who expected that they were not able to organise their daily life in such a way that they could participate in the exercise intervention twice a week. This expectation was based on the following reasons: the programme was too time consuming, they were too tired and they were unable to commit to the study and the exercise intervention. Secondly, 20% of the outpatients referred by a GP were admitted to the affective disorder programs after completing the intake procedure. However, by the time they were rated on the HAM-D17 (on average 10 weeks later), their score was already below 14, which made them ineligible to participate. Other causes for exclusion were medical contra-indication (25%) and already exercising twice a week for at least 30 min (15%). The planned dose of exercise was ultimately administered to 25 participants in the intervention group; the analysis was performed on a group of 24 participants (see Fig. 1). Between allocation to the intervention group (T0) and the first follow-up measurement (T3), 10 of the 25 initial participants (40%) dropped out. The drop-out rate increased to 56% at T6 and 80% at T12. In the control group, a similar drop-out pattern was seen: 43% at T3, 65% at T6 and 82% at T12 (Fig. 1). Of the 48 participants in both arms included in the study, two highly physically active participants (METS/week > 750 = extremely high PA) were excluded retrospectively from the analyses based on SQUASH outcomes. Ultimately, 46 remained for further analysis, with 24 participants in the intervention arm.

Due to low attendance at the blood lab (60% at T0 and 83% at T6), only the blood samples at T0 could be analysed.

### Participant characteristics at baseline

At baseline, 45% of the included participants were treated inpatients (17% were from the crisis care ward, 28% were from the day hospital) and 55% were outpatients. Table 1 shows the participant characteristics at baseline.

**Table 1:** Characteristics of the Intervention group, Control group and Total group^1^ at baseline. Mean (SD) unless noted otherwise

	Intervention group (*n* = 24)	Control group (*n* = 22)	Total group (*n* = 46)	Chi-square (^χ2^) Anova (F) Fisher’s exact (Fe)
Age, yrs.	42.2 (9.3)	40.1 (9.0)	41.2 (9.1)	F = .60
Sex, women %	79.2	40.9	60.9	^χ2^ = 7.05**
Education^2^, n	17	19	36	
Low %	18	26	22	Fe = .75
Middle %	47	53	50	
High %	35	21	28	
Hamilton-D17^3^	20.1 (5.2)	24.3 (5.4)	22.1 (5.6)	F = 7.33*
Fitness parameters^4^, n	24	20	44	
Wmax, Watt∙kg^− 1^	3.85 (.66)	3.91 (.65)	3.88 (.64)	F = 4.43*
VO_2_max, ml∙min^−1^∙kg^− 1^	29.1 (6.9)	35.7 (6.0)	31.6 (7.1)	F = 5.85*
Heartbeat at rest, b∙min^−1^	75.7 (12.3)	80.0 (16.0)	77.7 (12.2)	F = 1.10
Grip strength^5^, kg, n	18	16	34	
Left	31.3 (13.2)	33.6 (11.9)	32.4 (12.5)	F = .29
Right	33.1 (16.6)	38.6 (15.1)	35.4 (15.8)	F = .84
Body composition, n	24	22	46	
BMI	28.8 (5.9)	27.3 (6.1)	28.1 (6.0)	F = .70
Fat Free Mass, kg	51.2 (11.9)	58.5 (13.1)	54.7 (12.8)	F = 3.87
Muscle Mass, kg	27.0 (4.5)	31.1 (4.8)	29.0 (5.0)	F = 8.41*
Visceral Fat, kg	8.3 (2.8)	8.6 (4.5)	8.4 (3.6)	F = .08
Waist circumference, cm	98.3 (13.5)	97.4 (15.2)	97.9 (14.2)	F = .05
Blood pressure, n	24	22	46	
Systolic, mmHg	143.4 (24.2)	143.0 (17.1)	143.2 (20.9)	F = .00
Diastolic, mmHg	88.9 (12.5)	88.0 (9.6)	88.5 (11.1)	F = .07
Blood parameters^6^, n	18	10	28	
Fasting blood glucose, mmol∙l^−1^	4.9 (.44)	5.1 (.56)	5.0 (.48)	F = .598
Cholesterol, mmol∙l^−1^	5.1 (.68)	5.8 (1.33)	5.3 (.99)	F = 2.99
HDL Cholesterol, mmol∙l^−1^	1.33 (.36)	1.55 (1.04)	1.41 (.67)	F = .67
Cholesterol/HDL Cholesterol, mmol∙l^−1^	4.0 (.98)	4.7 (1.1)	4.4 (1.8)	F = 2.43
Triglycerides, mmol∙l^−1^	1.46 (.94)	1.8 (1.17)	1.5 (1.0)	F = .693
Anxiety: BAI^7^, n	10	11	21	
	18.5 (10.8)	28.5 (13.1)	23.7 (12.8)	F = 3.59
Pain: GCPS^8^, n	11	11	22	Fe = .58
A	2	1	3	
B	6	7	13	
C	3	3	6	
Pain medication %	64	72	68	F = .65
Quality of Life: EQ5-D^9^, n	17	21	38	
	37.5 (20.3)	29.7 (16.3)	33.2 (18.4)	F = 1.71
Costs: TIC-P^10^, n	19	21	40	
Contact GP last 6 weeks %	58	95	77	Fe = .007**
Work Sickness leave < 1 month %	85	85	85	Fe = .70
Work Sickness leave > 1 month %	54	54	54	Fe = .65
Housework disability %	71	77	74	Fe = .66
Housework help needed %	47	61	54	Fe = .52
Medication, n	24	22	46	
Antidepressant %	74	86	72	Fe = .44
Benzodiazepines %	42	68	50	Fe = .12
Mood stabilizer %	16	4	10	Fe = .32
Antipsychotic %	16	23	19	Fe = .70
Somatic medication %	31	45	39	Fe = .52
Duration antidepressant before inclusion				Fe = 1.0
0–3 months %	36	43	39	
3–6 months %	50	47	48	
> 6 months %	14	10	13	

* *p* < 0.05, ** *p* < 0.01 (2-tailed test)

^1^ 45% inpatients (17% emergency ward, 28% day-hospital), 55% outpatients

^2^ Missing questionnaires *n* = 10

^3^ 14–18: moderate depression, 19–27: severe depression, > 27: very severe depression

^4^ Two participants in the control group with physical problems during the bicycle test

^5^ Missing *n* = 12: Mechanical defect grip strength meter

^6^ Missing n = 22: No show at blood lab

^7^ Beck Anxiety Index, Missing *n* = 25; *16–26 moderate-severe anxiety, > 26 = severe anxiety*

^8^ Graded Chronic Pain Scale, Missing n = 24; Pain in body parts max = 7: A = 0–3, B = 3–5, C = 5–7

^9^ Euroquol 5-D, Missing *n* = 8. Subjective Health (1–100 scale, 100 = optimal)

^10^ Trimbos/iMTA Costs Associated with Psychiatric Illness, Missing *n* = 6

The baseline measurements showed that the intervention group included almost twice as many women than the control group, which has a large influence on parameters such as e.g. fitness (VO_2_max and Wmax) and muscle mass. The control group showed a significantly higher HAM-D17 score. In the total group, the criterion of hypertension (mean systolic blood pressure ≥ 140 mmHg) was met with a borderline mean diastolic blood pressure just below 90 mmHg. The mean BMI indicates overweight, and the mean waist circumference – a measure for central (visceral) adipositas – was above the norm: 95.0 (SD = 14.4) cm in females with ≥88 cm as cut-off score and 102.4 (SD = 13.3) in males with ≥102 cm as cut-off score, based on the criteria of the International Diabetes Federation (IDF) Epidemiology Task Force Consensus Group [38]. On the TIC-P, the control group had significantly more GP visits 6 weeks before inclusion. The majority of the participants were apparently treated along the depression protocol by a GP or mental health professional before inclusion, with on average 72% of the total group receiving antidepressants. Interpretation of the medication use indicated that some patients suffered from MDD with psychotic symptoms (melancholic depression) or/and were already in a following step of the MDD protocol (added lithium treatment). The severity of MDD was also underlined by the high mean rate of work sickness leave and inability to perform household tasks. The average delay between the intake procedure after GP referral, followed by an indication for the affective disorders programme until inclusion into the study for these outpatients, was 10.4 weeks. The participants in the emergency wards had to stabilise before inclusion, after which they were able to give informed consent and were included with an average delay of 3.5 weeks. As expected, due to the severity of their depression, the average HAM-D17 score at inclusion for inpatient or day hospital participants (25.7) was higher than for outpatients (20.5).

### Effectiveness of the intervention

Due to the high number of drop-outs, statistical analysis was performed for the primary outcome (HAM-D17), the submaximal Åstrand bicycle test (VO_2_max in ml∙min^− 1^∙kg^− 1^ and Wmax in W∙kg^− 1^), and the biometric values between T0 and T3 only. Intention to treat and per protocol analysis using GLM (interaction time by group) showed no significant additional reduction in depressive symptoms in favour of the intervention group. Depression on the HAM-D17 decreased in both groups on average by 2–3 points. Both fitness parameters of the submaximal Åstrand bicycle test, the BMI and visceral fat (the last one only in PP analysis) showed significant improvements compared to the control group. Although no significant difference between both groups was seen in waist circumference and visceral fat mass (in ITT analysis), these outcome variables showed a large clinical effect (Cohen’s d > 0.8) independent of the HAM-D17 scores. Heartbeat at rest and diastolic blood pressure, as a possible expression of autonomic nervous system modulation by exercise, showed a moderately large effect in favour of the intervention group (Table 2).

**Table 2:** Results of General Linear Model tests (interaction time by group) and effect sizes of Intention-to-treat (ITT) and Per-protocol (PP) analysis^1^

Outcome variable	Analysis	Intervention group T0	Intervention group T3	Control group T0	Control group T3	GLM (interaction time by group)	Effect size Cohen’s d
HAM-D17	ITT (NI = 12; NC = 7)	19.1 (5.2)	17.1 (6.6)	25.1 (6.6)	22.3 (8.3)	F = .13 p = .73	0.18
	PP (NI = 8; NC = 7)	17.7 (2.9)	14.4 (4.5)	25.1 (6.6)	22.3 (8.3)	F = .03 *p* = .85	0.10
Wmax (Watt.kg^−1^)	ITT (NI = 10; NC = 6)	2.29 (0.54)	2.64 (0.69)	2.76 (0.64)	2.73 (0.66)	F = 6.1 *p* = .03*	1.71
	PP (NI = 7; NC = 6)	2.24 (0.49)	2.60 (0.60)	2.76 (0.64)	2.73 (0.66)	F = 8.0 p = .02*	1.31
VO_2_max (ml∙min^−1^∙kg^− 1^)	ITT (NI = 10; NC = 6)	29.1 (6.8)	34.5 (8.6)	35.8 (6.0)	36.11 (7.9)	F = 7.1 p = .02*	1.47
	PP (NI = 7; NC = 6)	28.7 (6.1)	34.1 (7.2)	35.8 (6.0)	36.11 (7.9)	F = 14.3 *p* = .003**	2.29
Heartbeat at rest (b∙min^−1^)	ITT and PP (NI = 7; NC = 6)	90.0 (18.4)	79.4 (14.1)	82.1 (16.1)	84.8 (20.4)	F = 1.2 *p* = .30	0.65
BMI	ITT (NI = 12; NC = 6)	30.0 (6.0)	29.4 (7.1)	26.5 (6.6)	27.6 (6.9)	F = 5.0 p = .04*	1.19
	PP (NI = 8; NC = 6)	30.1 (6.2)	29.8 (6.9)	26.5 (6.6)	27.6 (6.9)	F = 6.0 p = .03*	1.42
Visceral Fat (kg)	ITT (NI = 12; NC = 6)	9.1 (2.4)	8.5 (3.1)	9.2 (5.3)	9.7 (5.0)	F = 4.1 *p* = .06	1.07
	PP (NI = 8; NC = 6)	8.9 (2.7)	8.5 (2.7)	9.2 (5.3)	9.7 (5.0)	F = 5.9 p = .03*	1.41
Muscle Mass (kg)	ITT (NI = 12; NC = 6)	28.4 (4.9)	29.2 (5.1)	33.6 (3.2)	33.3 (3.2)	F = 1.2 *p* = .27	0.58
	PP (NI = 8; NC = 6)	26.9 (4.3)	27.2 (4.2)	33.6 (3.2)	33.3 (3.2)	F = .77 *p* = .39	0.51
Waist circumference (cm)	ITT (NI = 12; NC = 7)	102.6 (10.7)	100.1 (10.5)	96.4 (18.6)	99.4 (18.3)	F = 3.4 *p* = .08	0.92
	PP (NI = 8; NC = 7)	102.4 (11.9)	100.4 (9.8)	96.4 (18.6)	99.4 (18.3)	F = 3.0 *p* = .11	0.96
Systolic Blood Pressure (mmHg)	ITT (NI = 12; NC = 7)	152.2 (23.4)	140.2 (18.6)	146.6 (22.7)	142.6 (17.9)	F = 1.2 p = .30	0.54
	PP (NI = 8; NC = 7)	143.5 (11.6)	138.0 (13.0)	146.6 (22.7)	142.6 (17.9)	F = .05 *p* = .82	0.12
Diastolic Blood Pressure (mmHg)	ITT (NI = 12; NC = 7)	90.9 (13.5)	81.7 (10.7)	86.3 (9.2)	83.9 (9.4)	F = 1.8 *p* = .19	0.67
	PP (NI = 8; NC = 7)	86.6 (11.4)	78.7 (9.5)	86.3 (9.2)	83.9 (9.4)	F = .95 *p* = .35	0.54

Data are mean (SD); NI = number in the intervention group; NC = number in the control group; GLM two-tailed test significance test: * = *p* < .05, ** = *p* < .01; Effect size (Cohen’s d): 0–20 = very small effect, >.20–.50 = small effect, >.50–.80 = moderate effect, >.80 = large effect (computed according method by Thalheimer & Cook, 2002). ^1^ PP ≥10 sessions attended

**Fig. 2:**
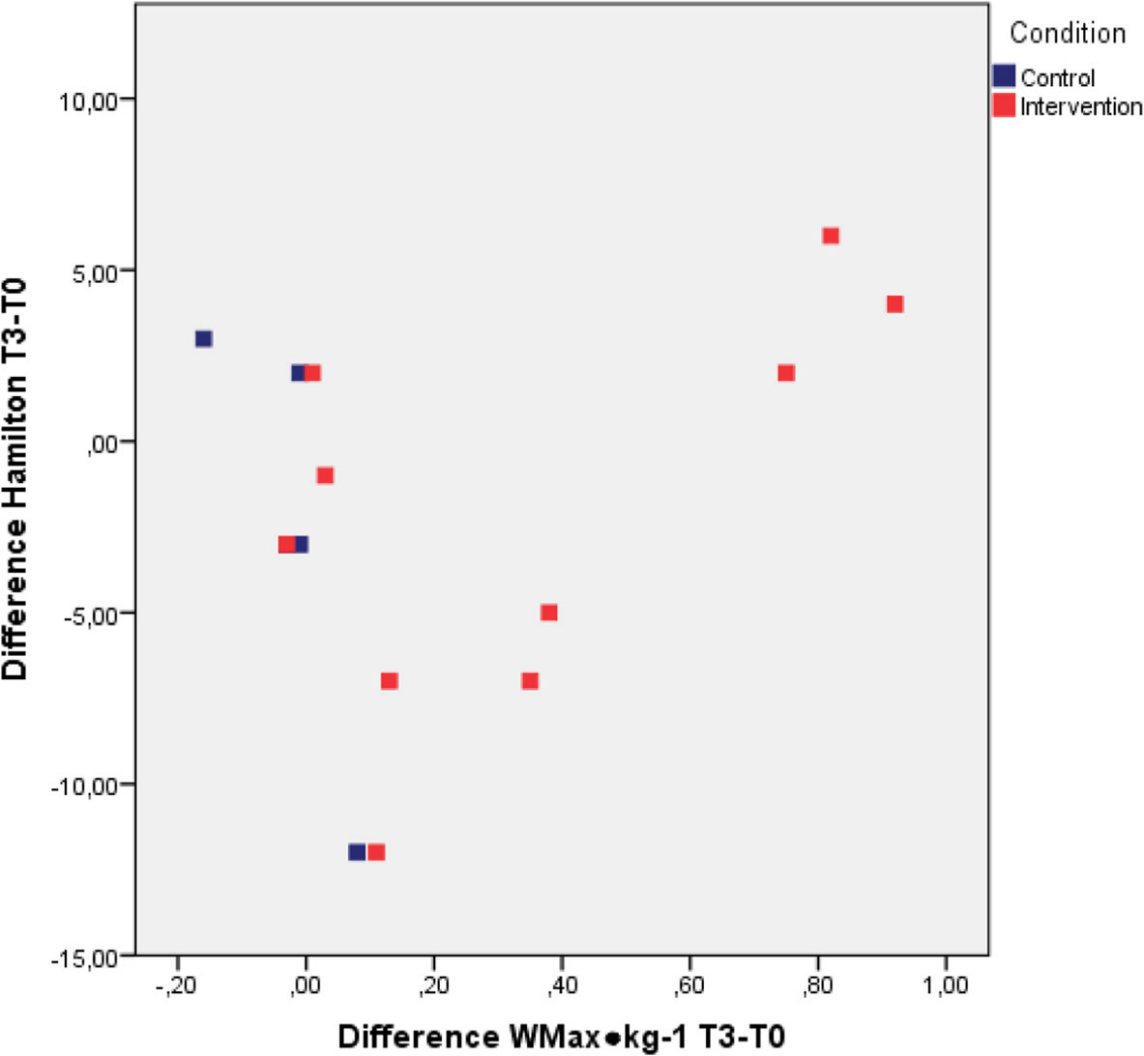
Intention-to-treat difference scores of the W∙kg^− 1^ plotted against difference scores of the Hamilton Rating Scale of Depression (HAM-D17), and labelled for intervention and control group

**Fig. 3:**
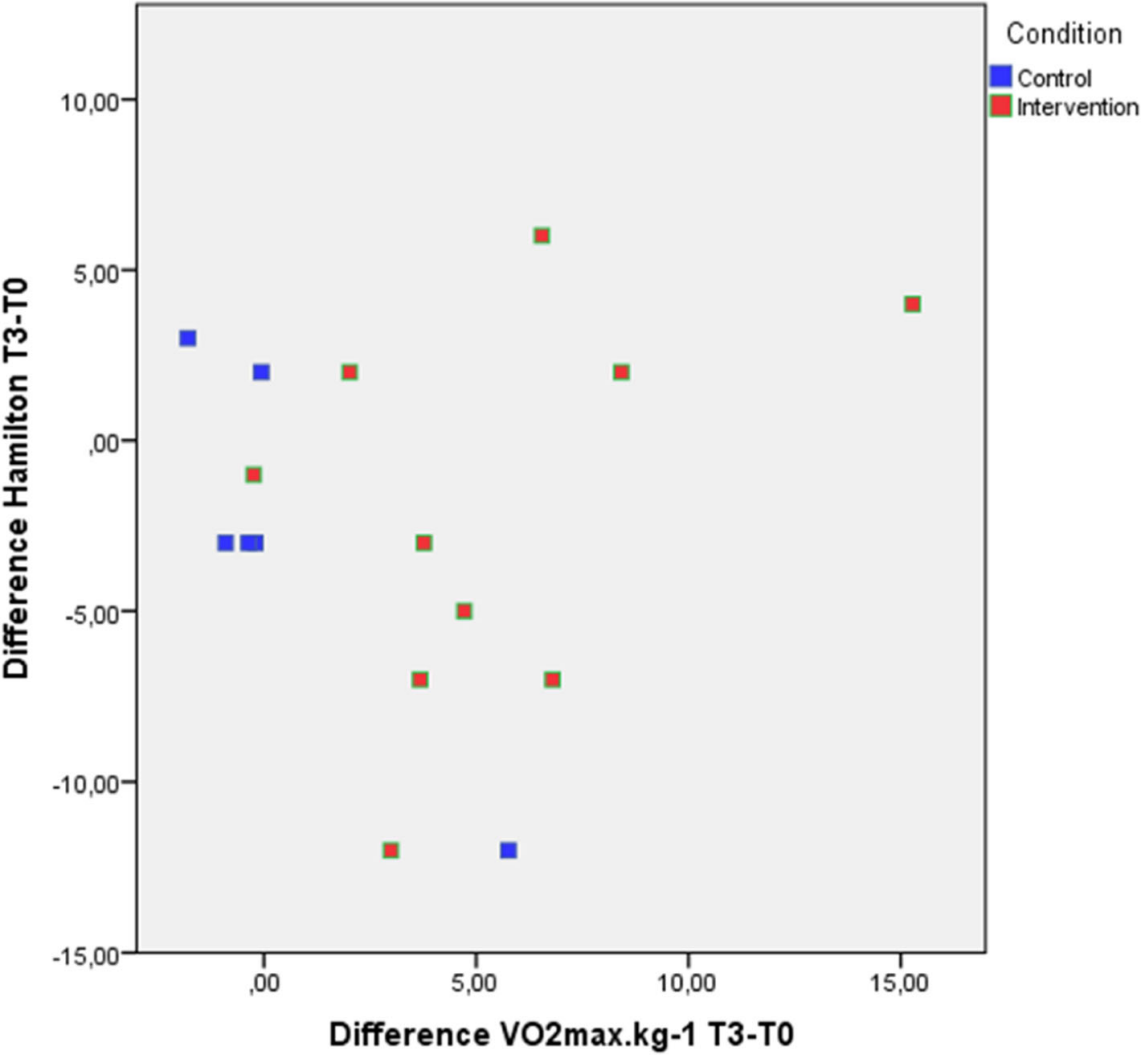
Intention to treat difference scores of the VO_2_max∙kg^− 1^ plotted against difference scores of the Hamilton (HAM-D17), and labelled for intervention and control group

For both the intervention and control group, Figs. 2 and 3 illustrate the difference in Wmax∙kg^− 1^ and VO_2_max∙kg^− 1^ between T3 and T0 in relation to the difference in HAM-D17 between T3 and T0. These figures show that the fitness of the intervention group increased greatly independent of the decrease in depression in both groups.

Analysis of effectiveness on blood fats, fasting glucose, BAI, GCPS, EQ-5D and TIC-P were not performed due to missing data.

## Discussion

The EFFORT-D RCT examined the effect of an add-on exercise intervention in patients with MDD treated in specialised mental health care in the Netherlands. To the best of our knowledge, this study is one of the few [16] RCTs with ‘real-life’ inpatients and outpatients with MDD which investigated the effect of an add-on aerobic exercise intervention on the course of depression. Moreover, few previous studies have used the evaluation of fitness parameters based on a submaximal Åstrand bicycle test [22, 23, 39], which is more objective and accurate than self-rated fitness [40].

Our main hypothesis – that a significant reduction in depressive symptoms would result from add-on exercise in the intervention group relative to the control group – was rejected. Both groups did improve at 3 months follow up, but there was no significant difference between the two arms. Given the small numbers of patients we were able to include, the lack of power precludes firm conclusions about the effectiveness of the intervention, and this RCT therefore can be characterised as a “failed trial”. However, the very small effect sizes (Cohen’s d was 0.18 in the ITT analysis and 0.10 in the PP analysis) suggest that a larger trial is unlikely to find a clinically relevant effect of add-on exercise on depression. The effects of the intervention on fitness (VO_2_max in ml∙min^− 1^∙kg^− 1^and Wmax in W∙kg^− 1^) were large, suggesting a considerable increase in fitness in the intervention group. The effects on metabolic risk factors, such as BMI, waist circumference and visceral fat, were large (Cohen’s d effect size > 0.8 in favour of the intervention group). This is in line with another recent 6 weeks add-on exercise study in MDD patients, in which physical fitness and metabolic syndrome factors improved in the exercise group [23]. A consistent component of significant changes in BMI and waist circumference in the EFFORT-D study is probably caused by a significant decrease of visceral fat. Given the relationship between white adipose tissue as a main player in the neuro-immune interactions between obesity and depression [41], a direct effect of exercise on the supposed chronic low-grade inflammatory state in these patients would be plausible. Unfortunately, lack of blood samples for CRP measurement precluded testing this. Regarding parameters reflecting a possible modulation of the autonomous nervous system, in which an overactive sympathetic activation is supposed to contribute to cardiovascular risk (CVR) factors [7], such as blood pressure and heartbeat at rest showed a moderately large effect size in the intervention arm.

A considerable proportion (40%) of the initially eligible depressed patients declined to participate in the study, and analysis of the drop-outs indicated low motivation to participate in this type of exercise intervention. Once included, adherence to the intervention was a main concern for our trial staff. Despite increasing effort in motivating participants and individual adaptations in exercise training, without violating the protocol, this tendency persisted. The fact that the number of drop-outs was equally high in both arms and that no inpatients completed the entire intervention for 3 months might be explained by the severity of MDD in this specific population. Low adherence to the exercise intervention is assumed to be associated with MDD symptoms such as fatigue, loss of energy and initiative [25]. A process evaluation [25] of the EFFORT-D study mainly pointed out that a lack of intrinsic motivation, assumed as part of the depressive symptomatology was a key factor in failing to continue the exercise. [42, 43]. This has been proposed as a potential key factor in the commencement and maintenance of PA in patients with severe mental disorders such as schizophrenia and affective disorders. In similar depressive populations as ours, two recent studies – add-on exercise compared to treatment as usual or two-armed exercise versus stretching RCTs – reported the same problems with inclusion and motivation [44, 45]. In these studies, 25 to 50% of the eligible participants lacked motivation to participate. In another recent pilot RCT examining the feasibility of an unsupervised, facility-based exercise programme in depressed patients, the most helpful aspect reported was connecting participants to fitness centre resources. Adding physical activity counseling as intervention however, did not increase exercise adherence and had no effect on depression scores [46]. This raises the question of whether this type and frequency of add-on exercise intervention, with also partly unsupervised sessions, is appropriate for patients with more severe MDD. Furthermore, it is very likely that RT and NW are not the preferred types of exercise for all patients in this population.

This suggests that exercise cannot be a standard intervention for all patients with MDD, and that more tailored interventions are needed [47]. The severity of MDD in this study population, together with its influence on several domains of daily life (somatic health, work, relations and social functioning), leads to the conclusion that an integrated lifestyle intervention appealing to these domains will probably be more effective than a single add-on exercise intervention. Therefore, more research is needed into potential negative influencers, patient profiling and for individually customised forms of exercise.

As a result of the 3-month exercise period, the fitness of the intervention group improved substantially: 15% increase in the Wmax. kg^− 1^ and 19% increase in VO_2_max. kg^− 1^. In another study in MDD patients, using 1 month of add-on exercise therapy and antidepressants (sertraline), an increase of 8% in cardiorespiratory fitness (VO_2_max) [48] combined with a reduction of sertraline use was found in the exercise group compared with the control group. The fact that less increase in cardiorespiratory fitness was found compared to our study could be explained by the shorter training period of 1 month. In addition, the baseline fitness of our participants was almost 10% lower relative to the fitness of an healthy but untrained Dutch working population [49], which could also play a role. This is because lower initial values leave more room for improvement. Our biometric findings in BMI, body composition (waist circumference and visceral fat) at baseline show a rather high risk of metabolic syndrome. This is in accordance with studies into the metabolic burden and CVR in affective disorders, in which a robust association was found between metabolic syndrome and MDD [2]. Patients with MDD are therefore assumed to be at high risk for cardiovascular morbidity and mortality. In contrast to earlier research indicating a high prevalence of diabetes Type II in MDD (DM-T2) [50], we found no indication of a high prevalence of diabetes Type II in the participants of the EFFORT-D study at baseline.

A recent meta-analysis showed a modest inverse dependent relationship between symptom severity and CVR, which was stronger in men than in women [51]. In the EFFORT-D study, however, the increase in Wmax∙kg^− 1^ and VO_2_max∙kg^− 1^ and was independent of the HAMD-17 results. Furthermore, a recent population based meta-analysis showed the protective effect of PA on depression [52]. The question remains whether this protective effect also applies to patients who recently remitted from a depressive episode.

In closing, although our primary hypothesis was rejected in this RCT, our results showed a potentially lower cardiovascular risk profile as a result of the intervention, which is a benefit when considering the high cardiovascular risk of depressive patients.

### Limitations and strengths

The most important limitation of this RCT is that only about 25% of the intended participants were included, which clearly had a negative impact on the power of the study. The underlying reasons were described in a separate process evaluation paper [25]: a lack of motivation of the patients to participate (or continue to participate) in the study and a dynamic reorganizational surrounding (budgetary cuts and a merger) were the main causal factors. The lack of motivation was assumed to be related to the severity of the patients’ depression in combination with (based on the patient records) the psychosocial circumstances which caused and maintained the depressive episode. In addition, excessive loss to follow-up made it impossible to perform the intended statistical analysis of follow-up data at 6 and 12 months. However, the non-significant result of the additional exercise intervention on depression that we found after 3 months of exercise in this type of patients is in accordance with a recent robust meta-analysis in this field, with DSM and ICD diagnosed MDD patients [53].

A further limitation was insufficient data for the analysis of several laboratory parameters and the results of questionnaires on quality of life, anxiety and pain symptoms, and cost-effectiveness. For example, due to the high proportion of no-shows at the blood lab, it was impossible to compare metabolic and inflammatory factors in the blood samples at T0 and T6, as intended in the design of the EFFORT-D study.

One strength of this study is its high-quality design: it was a RCT using the semi-structured Hamilton interview to diagnose the severity of MDD and the Åstrand submaximal bicycle test to objectively measure physical fitness. In addition, the EFFORT-D study is one of the few studies into MDD in ‘real-life’ patients, offering a useful new clinical perspective of the role of exercise in MDD. The objective measurement of the fitness parameters using bicycle-testing instead of self-rating by patients may also contribute to more scientific evidence for CVR in MDD [54] and prevention in the future.

### Clinical implications

This RCT showed that low motivation for exercise and low adherence after starting exercise greatly reduces the number of MDD patients eligible for an add-on intervention. Given the non-significant results on depression scores, the clinician is confronted with the question of whether RT or NW are still considered positive interventions in ‘real-life’ patients in specialised mental health care. Our data on health parameters, however, showed a significant effect on risk factors for cardiovascular disease, so it seems logical to emphasise these positive health effects in psycho-education to motivate patients to exercise instead of promising them reduction of depressive symptoms on the short term. This might prevent even more feelings of failure in clinical patients, which are already a burden related to their MDD.

## Recommendations

Future studies into exercise for MDD patients should focus on participation from the first moment of contact with the patient. Risk factors for drop-out and low adherence are increasingly known and could be identified in individual patients, thus offering a risk-profile leading to more tailored attention to and guidance of the patient. In addition, more research is needed to identify factors that precipitated non-adherence. Participation might also be improved by using electronic devices (such as pedometers or actometers), social media and visits to the patients’ homes (agreed to in advance). Furthermore, with regard to the response to the questionnaires, the online versions were not very appropriate for the MDD patients in the EFFORT-D study, leading to many incomplete questionnaires. Since all measurements performed in the presence of the research assistant or blind outcome assessors were more complete, filling in questionnaires in the presence of a trial staff member is recommended instead of self-rating online versions at home.

## Conclusions

The current study underlines the importance of an add-on exercise intervention in MDD patients to reduce their cardiovascular risk by enhancing their fitness. For inpatients and day-hospitalised patients with MDD, and those with a potentially more resistant depressive symptoms or with a higher cardiovascular risk, exercise could prevent future health problems. Due to a low statistical power and lack of follow-up at six and 12 months, conclusions about the anti-depressive effect of this add-on exercise intervention were not possible. However, the effect size on depression was so low that even a higher number of participants would not have improved the effect-size to clinical relevance. This is in line with results of previous meta-analyses when the results of the methodologically weaker studies are excluded. It could be more appropriate for clinicians to psycho-educate their MDD patients and to emphasise the useful effects of exercise on cardiovascular risk factors, rather than promising them unlikely positive results on depressive symptoms in the short term.

## Data Availability

The data that support the findings of this study are not publicly available because of the inconsistency with the informed consent. However, anonymous data are available from the corresponding author on request and with permission of GGZ Centraal.

## Abbreviations

(M)ANOVA: (Multivariate) Analysis of Variance
BAI: Beck’s Anxiety Inventory
BMI: Body Mass Index
CRP: C-reactive Protein
CVR: Cardio Vascular Risk
DM: Diabetes Mellitus
EFFORT-D study: Effect of Running on Depression study
EQ-5D: EuroQol Health Questionnaire – 5 Dimensions
FE: Fisher’s Exact Test
GCPS: Graded Chronic Pain Scale
GLM: General Linear Model
GP: General Practitioner
HAM-D 17: 17-item Hamilton Depression Rating Scale
HDL/LDL: High/Low Density Lipoproteins
IDS-SR: Inventory of Depressive Symptomatology - Self Rating
ITT: Intention to treat analysis
LEQ: Life Events Questionnaire
MDD: Major Depressive Disorder
MET: Metabolic Equivalent
NW: Nordic Walking
PP: Per protocol analysis
RCT: Randomised Controlled Trial
RT: Running Therapy
SD: Standard deviation
SQUASH: Short Questionnaire to Assess Health Enhancing Physical Activity
T0, T3, T6, T12: Measurements at inclusion, three, six and 12 months
TIC-P: Manual Trimbos/iMTA Questionnaire for Costs Associated with Psychiatric Illness
VO_2_max: Maximal oxygen uptake
WHO-DAS: World Health Organisation - Disability Assessment Schedule
Wmax: Maximal external output
